# Verantwortungsvoller Einsatz von KI und Robotik in der Medizin: Chancen, Herausforderungen und Perspektiven für die HNO-Heilkunde

**DOI:** 10.1007/s00106-025-01716-y

**Published:** 2026-01-15

**Authors:** Florian Funer

**Affiliations:** Institut für Ethik und Geschichte der Medizin, Eberhard-Karls-Universität Tübingen, Gartenstr. 47, 72074 Tübingen, Deutschland

**Keywords:** Ethik, Digitalisierung der Medizin, Verantwortung, Transparenz, Legitimation, Ethics, Digital transformation in medicine, Responsibility, Transparency, Legitimation

## Abstract

Künstliche Intelligenz (KI) und Robotik verändern die Grundlagen medizinischer Praxis. Ihre Integration eröffnet neue Chancen für Präzision, Individualisierung und Effizienz, wirft jedoch zugleich grundlegende ethische Fragen nach Wissen, Verantwortung und Legitimation auf. Der Beitrag fasst zentrale Chancen und Risiken des Einsatzes von KI und Robotik in der Medizin zusammen und zeigt, wie sich durch diese Technologien epistemische, normative und soziale Dimensionen ärztlicher Praxis verschieben. Zwar bietet die Vielzahl bestehender ethischer Prinzipien und Leitlinien eine erste Orientierung, doch erst durch ihre Einbettung in konkrete klinische, institutionelle und prozessgeleitete Strukturen entfalten diese praktische Wirksamkeit. Drei Leitideen werden als Grundlage einer solchen Ethik vorgeschlagen: epistemische Transparenz, geteilte Verantwortung und diskursiv-prozedurale Legitimation. Sie bilden die Grundlage einer lernenden, reflexiven und patientenzentrierten Ethik des verantwortungsvollen Technologieeinsatzes.

## Abstract (en)

Artificial intelligence (AI) and robotics are transforming the foundations of medical practice. Their integration creates new opportunities for precision, individualization and efficiency but also raises fundamental ethical questions about knowledge, responsibility and legitimacy. This article summarizes the main opportunities and risks of the use of AI and robotics in medicine and shows how these technologies reshape the epistemic, normative and social dimensions of medical practice. While numerous ethical principles and guidelines provide initial guidance, the real impact arises only when the principles are embedded in concrete clinical, institutional and procedural contexts. There are three guiding ideas that are proposed as the basis of such ethics: epistemic transparency, shared responsibility and discursive-procedural legitimacy. These form the foundation of a learning, reflective and patient-centered ethics of the responsible use of technology.

## Einführung

Künstliche Intelligenz (KI) und robotische Systeme prägen zunehmend die klinische Praxis. Sie werden in der Diagnostik, in der Therapieplanung und in operativen Verfahren eingesetzt und verändern dabei die Abläufe des medizinischen Handelns oftmals wesentlich. Während technologische Innovationen die Medizin seit jeher begleiten, markiert die Integration lernender und adaptiver Systeme einen qualitativen Wandel. KI und Robotik sind nicht länger bloß Instrumente, sondern umstrukturierende Systeme, die auf Basis von Daten und Mustererkennung Vorschläge generieren, priorisieren und auf diese Weise Entscheidungen unterstützen – mitunter leiten – sollen. Dadurch entstehen neue Fragen nach Wissen, Verantwortung und normativer Orientierung der medizinischen Praxis.

Ethische Reflexion ist in diesem Zusammenhang unverzichtbar, weil mit der Digitalisierung und Automatisierung nicht nur neue technische Möglichkeiten entstehen, sondern auch die normativen Grundlagen des ärztlichen Handelns verschoben werden können. Medizinisches Handeln beruht heute üblicherweise auf Prinzipien wie Fürsorge, Nicht-Schaden, Gerechtigkeit sowie Respekt vor der Autonomie der Patientinnen und Patienten [1]. Wenn Entscheidungsfindung zunehmend durch algorithmische Modelle und robotisierte Hilfsmittel unterstützt wird, deren innere Logik weder nachvollziehbar ist noch intentionale Handlungen von Vertrauenspersonen abbildet, stellt sich die Frage, wie diese Prinzipien in einer digitalisierten Medizin weiterhin tragfähig bleiben können und für das Handeln in der medizinischen Praxis Orientierung geben sollen.

Diese Herausforderungen betreffen die Medizin in ihrer gesamten Breite, wenn sie sich auch in verschiedenen Anwendungsfeldern in unterschiedlichem Ausmaß manifestieren mögen. Die Hals-Nasen-Ohren-Heilkunde (HNO) kann dabei als exemplarisches Anwendungsfeld dienen, an dem sich die Dynamiken der Technologisierung und Digitalisierung paradigmatisch beobachten lassen (vgl. hierzu u. a. auch die Überblicksarbeiten in dieser Zeitschrift [2, 3]). Die folgenden drei Beispiele des Einsatzes illustrieren diese Entwicklungen:

1. *Radiomics* ermöglicht die KI-gestützte Auswertung bildgebender Daten, bei der aus CT(Computertomographie)-, MRT(Magnetresonanztomographie)- oder PET(Positronenemissionstomographie)-Scans hunderte quantitative Merkmale extrahiert und z. B. mit histopathologischen und molekularbiologischen Tumoreigenschaften korreliert werden. In der Kopf-Hals-Onkologie konnte so etwa die HPV(humane Papillomaviren)-Assoziation oropharyngealer Plattenepithelkarzinome oder die Expression von Immuncheckpoint-Molekülen wie PD-L1 („programmed death-ligand 1“) aus PET/CT-Daten mit hoher prognostischer Genauigkeit vorhergesagt werden, wodurch sich Perspektiven einer nicht-invasiven, datenbasierten Tumorcharakterisierung eröffnen [2].
2. Die *transorale roboterassistierte Chirurgie *ermöglicht die minimal-invasive Entfernung von Oropharynxkarzinomen über den Zugang des Mundraums. Durch die dreidimensionale (3-D) Visualisierung und hochgradig präzise Instrumentensteuerung lassen sich Tumoren auch in anatomisch schwer zugänglichen Regionen resezieren, wodurch postoperative Erholungsphasen und funktionelle Beeinträchtigungen (z. B. Schlucken) im Vergleich zu offenen Verfahren reduziert werden könnten [3].
3. *KI-gestützte Diagnostik in der Otologie und Neurootologie* wiederum nutzt KI-Modelle zur Auswertung ohrmikroskopischer Aufnahmen, audiologischer Daten oder okulomotorischer Befunde. Auf diese Weise lassen sich etwa Trommelfellbefunde aus otoskopischen Bildern mit einer Genauigkeit von beinahe 90 % klassifizieren, das Hörergebnis nach Hörsturz anhand audiometrischer Variablen prognostizieren und Nystagmusmuster bei benignem paroxysmalen Lagerungsschwindel dem betroffenen Bogengang korrekt zuordnen [2].

Diese Beispiele verdeutlichen, dass KI und Robotik in der Medizin zunehmend in diagnostische, therapeutische und rehabilitative Prozesse integriert werden können und dort neue Handlungsmöglichkeiten schaffen. Freilich ist zu betonen, dass die möglichen Anwendungsbereiche dieser Technologien weit über die klassische ärztliche Praxis hinausreichen. Auch Pflege, Administration und Public Health nutzen zunehmend KI-basierte und etwas seltener roboterassistierte Systeme (vgl. hierzu z. B. [4]). Der vorliegende Beitrag konzentriert sich jedoch bewusst auf den medizinisch-ärztlichen Kontext, der von aktuellen Innovationen besonders häufig betroffen ist und in dem sich Fragen nach Verantwortung, Wissen und Entscheidungsfindung verdichten. An ihnen lässt sich aufzeigen, dass KI und Robotik nicht lediglich technische Erweiterungen bestehender Verfahren darstellen, sondern dass mit ihrem Einsatz auch eine Transformation der medizinischen Praxis in mindestens dreifacher Hinsicht einhergehen kann:

- *Epistemisch* (d. h. in Bezug auf die Entstehung und Rechtfertigung medizinischen Wissens) verändern sie, wie medizinisches Wissen entsteht und bewertet wird sowie welche Instanzen als erkenntnisleitend gelten. Ärztliche Erfahrung und algorithmische Mustererkennung treten in ein neues Verhältnis, das Fragen nach Verlässlichkeit und Nachvollziehbarkeit aufwirft (vgl. [5, 6]).
- *Normativ* (d. h. im Hinblick auf die Maßstäbe, nach denen Entscheidungen als moralisch gut, richtig oder verantwortungsvoll gelten) beeinflussen sie, was als angemessene medizinische Entscheidung gilt und wie diese verantwortet wird. Kriterien wie Präzision und Effizienz gewinnen womöglich an Gewicht, während Werte wie Erfahrungswissen oder klinische Urteilskraft neu zu verorten sind (vgl. [7, 8]).
- *Sozial* (d. h. bezogen auf die institutionellen und interpersonellen Strukturen sowie Interaktionen des medizinischen Handelns) verändern sie die Rollen und Interaktionen zwischen Ärztinnen und Ärzten, Patientinnen und Patienten und Institutionen sowie deren Strukturierung von Entscheidungsprozessen. Verantwortung und Autorität werden neu verteilt, und die Erwartungshorizonte von Beteiligten verschieben sich (vgl. [5, 9]).

Neben der technischen Analyse von Leistungsfähigkeit und Sicherheit, der klinischen Bewertung von Wirksamkeit und Nutzen, der gesundheitsökonomischen Betrachtung von Kosten und Ressourceneffizienz sowie der regulatorischen Prüfung von Konformität und Zulassung bedarf es daher einer ethischen Analyse, die diese Dimensionen ergänzt und kritisch vertieft. Sie fragt, wie sich durch den Einsatz von KI und Robotik die Grundlagen verantwortlichen Handelns verändern und wie Rechenschaft, Transparenz und Fürsorge unter digitalen Bedingungen gestaltet werden können.

Erfreulicherweise hat sich parallel zur zunehmenden Implementierung digitaler Systeme eine umfassende ethische und regulatorische Diskussion entwickelt. Zahlreiche Publikationen und Governance-Initiativen formulierten bereits Prinzipien und Rahmenbedingungen v. a. für einen verantwortungsvollen KI-Einsatz in der Medizin (vgl. hierzu etwa [10–13] und überblickhaft [14]). Dennoch zeigt sich, dass die Herausforderung weniger in einem Mangel an oder einem zeitlichen Rückstand der Ethik liegt, sondern in ihrer Anwendung: Prinzipien bleiben oftmals zu allgemein, zu formalisiert oder zu weit von den konkreten klinischen Abläufen entfernt, um die Implementierung von Technologien und klinische Entscheidungsprozesse wirksam zu leiten. Dadurch entsteht eine Lücke zwischen ethischer Reflexion und medizinischer Realität. Prinzipien existieren, werden aber nicht operationalisiert – sie bieten Orientierung auf dem Papier, ohne jedoch die Interaktionen und Verantwortlichkeiten im klinischen Alltag wirklich zu strukturieren.

Der gegenwärtige Moment ist daher ein entscheidender. Während Entwicklungen und Erprobungen mancherorts bereits weit fortgeschritten sein mögen, bestehen nach wie vor weitreichende Chancen, ihre klinische Implementierung und systemische Nutzung aktiv zu gestalten. Ziel dieses Beitrags ist es, zu dieser Gestaltung beizutragen – durch eine überblickhafte Darstellung der zentralen Chancen und Risiken, eine Reflexion der strukturellen Transformationen der medizinischen Praxis sowie die Entwicklung von Leitideen für einen prozess- und kontextgeleiteten verantwortungsvollen Einsatz von KI und Robotik in der Medizin.

## Chancen und Risiken von KI und Robotik – eine Diskursübersicht

Die Einführung von KI und Robotik in der Medizin eröffnet eine Reihe von Chancen und Risiken für die medizinische Praxis. Im Folgenden werden einige der zentralen ethischen Aspekte skizziert, die die Literatur zur Ethik dieses Technikeinsatzes bislang dominierten (vgl. z. B. [15–17]). Die Übersicht erhebt keinen Anspruch auf Vollständigkeit, sondern soll exemplarisch die ethischen Dimensionen und Implikationen aufzeigen, die beim Einsatz dieser Technologien relevant sind. Viele hiervon bedingen sich gegenseitig und überlappen mitunter.

Die folgenden Chancen und Risiken zeigen, dass der Einsatz von KI und Robotik in der Medizin erhebliche ethische und praktische Spannungsfelder eröffnet. Erkennbar wird dabei, dass häufig dieselben Eigenschaften die ihren Einsatz potenziell problematisch machen können, wie ihre Leistungsfähigkeit, Komplexität und operative Eigenständigkeit, auch Ausgangspunkt ihres möglichen Nutzens für eine bessere Gesundheitsversorgung sind.

### Risiken und Bedarfe der Regulierung

#### Beurteilung von Wirksamkeit und Zuverlässigkeit

Die Bewertung der Wirksamkeit und Zuverlässigkeit lernender und robotischer Systeme folgt vielerorts bislang nicht einem Standard, der den etablierten, strikten Anforderungen evidenzbasierter Medizin entspricht. Zwar unterliegen KI-Anwendungen und robotische Systeme formal dem europäischen Medizinprodukterecht und benötigen für ihren breitenwirksamen Einsatz eine CE(Conformité Européenne)-Kennzeichnung, doch die zugrunde liegende Konformitätsbewertung unterscheidet sich grundlegend von der Zulassung eines Arzneimittels. Sie verlangt in der Regel keinen Nachweis klinischer Wirksamkeit durch randomisierte, kontrollierte Studien, sondern lediglich den Beleg technischer Sicherheit und funktionaler Leistungsfähigkeit. Damit kann ein System als regelkonform gelten, ohne dass belegt wäre, ob und in welchem Umfang es tatsächlich einen medizinischen Nutzen bringt.

Belastbare Evidenz entsteht hier noch deutlicher als bei Medikamenten aus der Interaktion von Mensch, Technik und Kontext, also aus einem hybriden System, dessen Leistung sich weder auf algorithmische Präzision noch auf mechanische Genauigkeit reduzieren lässt. Randomisierte Studien stoßen jedoch da an methodische Grenzen, wo technische Systeme und der Erfolg ihres Einsatzes wesentlich von der Nutzungspraxis abhängen – auch hierin dürfte der Mangel solcher randomisierten, prospektiven Studien in der HNO-Heilkunde (vgl. z. B. [3]) begründet liegen. Darüber hinaus bedürfte jedes Update von Software, das nach der Zulassung weiter angepasst oder trainiert werden kann, einer erneuten klinischen Prüfung, um eine Evidenzlücke zwischen regulatorischer Zulassung und realer Wirksamkeit zu verhindern. Diese und weitere Unterschiede stellen die gegenwärtige Evaluations- und Begutachtungspraxis vor Herausforderungen [18].

Ethisch entscheidend ist daher, dass Validierung nicht nur als technischer Nachweis verstanden wird, sondern als Frage nach epistemischer und klinischer Verlässlichkeit. KI und Robotik müssen in den Bedingungen geprüft werden, unter denen sie tatsächlich eingesetzt werden sollen – im Zusammenspiel mit ärztlicher Erfahrung, organisationalen Strukturen und Patienteninteraktionen. Nur eine solche „ökologische“ Evaluation kann zeigen, ob ein System nicht nur *in silico* korrekt funktioniert, sondern auch *in praxi* vergleichbar oder gar besser wirksam ist.

#### Biases, Diskriminierung und das Problem der Gerechtigkeit

Das bekannte Problem oftmals mangelnder Repräsentativität von Datensätzen kann angesichts der induktiven und probabilistischen Funktionsweise insbesondere KI-gestützter Anwendungen dazu führen, dass bestehende Ungleichheiten reproduziert oder gar verstärkt werden. Derartige Systeme zeigen daher regelmäßig eine ungleiche Leistungsfähigkeit für unterschiedliche Gruppen, etwa hinsichtlich Geschlecht, Alter, Hautfarbe oder sozioökonomischem Status [11, 12]. Solche Unterschiede werden insbesondere dann ethisch problematisch, wenn sie auf sog. geschützte Merkmale zurückgehen und damit auf Kategorien, in denen Menschen besonders häufig strukturell benachteiligt werden.

Die Diskussion über Bias und Gerechtigkeit betrifft nicht nur KI-Systeme, sondern auch robotische Anwendungen, etwa wenn Operationsroboter nur für standardisierte Körperproportionen kalibriert sind. Diese Formen des algorithmischen oder technischen Bias sind selten Ausdruck von Intention, sondern das Ergebnis ungleicher Datengrundlagen, unausgewogener Modellarchitekturen oder – etwas voraussetzungsreicher – impliziter Annahmen über den „Normalfall“ des menschlichen Körpers oder dessen Verhalten. Der Umstand, dass Biases bei datenbasierten Technologien auftreten und im Falle ihres systematischen Einsatzes weitreichende nachteilige Konsequenzen für unterrepräsentierte Personengruppen haben können, sollte dabei jedoch nicht darüber hinwegtäuschen, dass Verzerrungen und darauf basierende Praktiken auch bei bereits etablierten medizinischen Maßnahmen vorkommen. Wenn auch in ihrer Tragweite noch nicht immer in die Praxis übersetzt, sind heute etwa unterschiedliche Effekte von Arzneimitteln bei Personen unterschiedlichen Geschlechts oder unterschiedlichen Gewichts offenkundig. Dennoch sind womöglich ebenso relevante Unterschiede zwischen anderen Personengruppen der Medizin heute noch weithin unbekannt.

Ungerechtigkeit besteht da, wo vergleichbare Fälle ohne moralisch relevanten Grund ungleich oder nicht vergleichbare Fälle ohne moralisch relevanten Grund gleich behandelt werden. Ethisch verlangt dies im Falle datenbasierter Technologien eine doppelte Aufmerksamkeit: Zum einen müssen Datensätze und Modelle so gestaltet werden, dass Diversität und Repräsentativität der Daten systematisch gesichert sind. Zum anderen muss transparent werden, wie und für wen ein System zuverlässig funktioniert. Unterschiedliche Ansätze zum Umgang mit diesem Problem – von der Korrektur von Trainingsdaten über gezielte Aufklärung der betroffenen Gruppen bis hin zur bewussten Einschränkung der Nutzung auf Kontexte, in denen ausreichend Daten gewährleistet sind – werden diskutiert, deren Umsetzung in der Regel mit unterschiedlichem Ressourcenaufwand verbunden ist.

Mit zunehmender Individualisierung, etwa im Rahmen der Präzisionsmedizin (vgl. weiter unten), entstehen jedoch auch neue Spannungsfelder. Je feiner die Gruppen, auf deren Basis Entscheidungen getroffen werden, desto kleiner wird die Datenbasis für diese konkrete Entscheidung, auf der ein System trainiert wurde. So droht der Gewinn an Präzision zugleich ein Verlust an Verlässlichkeit zu werden. Diesen „trade-offs“ der Leistungsfähigkeit von Robotik und KI liegt eine genuin ethische Frage zugrunde: Ist die Inkaufnahme von mehr Fehlern für eine Personengruppe angesichts des großen Nutzens für eine (vermutlich) größere Personengruppe moralisch legitim und wenn ja, unter welchen Bedingungen? Die Antwort auf diese Frage wird je nach zugrunde gelegter Vorstellung von Gerechtigkeit anders ausfallen. Eine verantwortungsvolle Praxis steht hier vor der Aufgabe, Verteilungs- und Teilhabegerechtigkeit als integrale Dimension technologischer Entwicklung von Anfang an zu berücksichtigen und in Designentscheidungen zu übersetzen.

#### Normative Setzungen der Technik und fehlende Wertflexibilität

KI und Robotik sind keine wertneutralen Werkzeuge. Sie müssen auf bestimmte Ziele hin entwickelt und trainiert werden – und jedes Ziel setzt voraus, dass zuvor entschieden wurde, was als wertvoll und erstrebenswert gilt [5, 19]. In der Medizin sind diese Werte häufig geteilt, etwa Lebensverlängerung, Schmerzfreiheit oder Verbesserung der Lebensqualität. Doch selbst hier bestehen Spannungen, etwa zwischen Dauer und Qualität des Lebens, zwischen Funktionserhalt und Ganzheitlichkeit oder zwischen individueller Präferenz und systemischer Effizienz.

Die Entwicklung entscheidungsleitender technischer Systeme zwingt dazu, Werte explizit zu operationalisieren. Wer ein Modell trainiert, legt fest, welche Parameter als Ziele verfolgt werden, und damit, welche „Formen des Guten“ technisch reproduziert werden. In der Onkologie kann dies die Priorisierung von Überlebenszeit gegenüber Lebensqualität bedeuten, in der Robotik etwa die Gewichtung von Ressourcenschonung gegenüber ästhetischen oder funktionellen Aspekten. Jede technische Entscheidung ist somit auch eine ethische Entscheidung, weil sie eine normative Ordnung in digitalen Code und technisches Design übersetzt.

Ethisch verantwortliche Technikgestaltung verlangt daher Werttransparenz und die Anerkennung von Wertpluralismus [5, 7]: Systeme müssen so konzipiert und implementiert werden, dass sie unterschiedliche Vorstellungen vom Guten zulassen und diese in klinischen Entscheidungsprozessen reflektiert werden können [19].

#### Autonomie und Entscheidungsfindung

Die zunehmende Integration von KI und Robotik in die klinische Entscheidungsfindung berührt unmittelbar die Frage nach ärztlicher und patientenseitiger Autonomie [5, 7, 8, 9 ]. Während die Medizin sich in den vergangenen Jahrzehnten bewusst vom paternalistischen Modell des „Arztes als Entscheider im besten Interesse des Patienten“ entfernt hat, droht durch lernende und automatisierte Systeme eine subtile Rückkehr des Paternalismus – diesmal in technischer Gestalt.

Maschineller Paternalismus [9, 19] entsteht, wenn Systeme Behandlungsoptionen aufgrund impliziter Wertentscheidungen (vgl. oben) priorisieren, ohne die individuellen Präferenzen der Betroffenen zu berücksichtigen. Wenn diese Wertentscheidungen unsichtbar bleiben und nicht an die Ansprüche des individuellen Patienten bzw. der individuellen Patientin angepasst werden können, wird Autonomie untergraben, nicht weil jemand ihr aktiv zuwiderhandelt, sondern weil sie gewissermaßen still vorausgesetzt und technisch fixiert wird [5].

Ein verantwortungsvoller Einsatz erfordert daher Transparenz über die Wertgrundlagen der Systeme und ihre Kommunikation im gemeinsamen Entscheidungsfindungsprozess. Ärztinnen und Ärzte müssen verstehen, welche Ziele durch den Einsatz eines Systems verfolgt werden, um dessen Empfehlungen in die individuellen Wert- und Präferenzstrukturen der Patientinnen und Patienten einordnen zu können [5, 6]. Autonomie hat daher nicht notwendigerweise zur Folge, dass der Einsatz von Technik ausgeschlossen wird, sondern dass Einsicht sowie dadurch Kontrolle und Mitgestaltung gewahrt bleiben [7].

#### Verantwortung und Haftung

Verantwortung im medizinischen Handeln hängt rechtlich wie ethisch vorrangig von 2 Faktoren ab, nämlich vom Grad der Kontrolle über das Ergebnis und von der Vorhersehbarkeit der Folgen [8]. Wird ein Schaden verursacht, stellt sich die Frage, wer ihn hätte verhindern können und wer über das nötige Wissen verfügte, um das Risiko zu erkennen. Dieses Prinzip kann durch KI und Robotik grundlegend herausgefordert werden, da sich die Kontrolle über klinische Ergebnisse durch den Einsatz von Technologien zunehmend auf Mensch, Maschine und Institution verteilt.

In der ärztlichen Praxis bleibt die (Letzt‑)Verantwortung zunächst bei der behandelnden Person [10, 11], die das System auswählt, dessen Ergebnisse interpretiert und in der Regel anwendet. Sie ist verpflichtet, sich über Leistungsdaten, Grenzen und Risiken der eingesetzten Technologie zu informieren sowie Patientinnen und Patienten über Nutzen und Unsicherheiten aufzuklären. Dennoch kann keine Ärztin für Fehler verantwortlich gemacht werden, die aus nicht vorhersehbaren technischen Fehlfunktionen oder aus undurchschaubaren KI-Prozessen resultieren, die sich dem Einfluss entziehen. Robotische Systeme und KI-basierte Entscheidungsunterstützung erzeugen somit hybride Verantwortungsarchitekturen [8]. Neben die klinische Verantwortung treten die Pflichten jener, die für Design, Sicherheit, Validierung und kontinuierliche Überwachung verantwortlich sind, ebenso wie jener, die über den konkreten Einsatz eines technischen Systems und die Implementierung von Standards innerhalb einer Institution entscheiden. Auch sie tragen moralische Verantwortung, wenn Werte, Zielkriterien oder Grenzen der Systeme unzureichend offengelegt werden oder die Systeme mangelhaft getestet sind.

Ethisch entscheidend ist nicht, Verantwortung zu verschieben, sondern sie strukturiert zu teilen. Dies erfordert klare Rollen, nachvollziehbare Zuständigkeiten und transparente Rechenschaftswege. Eine solche Verantwortungskultur verlangt, dass Ärztinnen in der Nutzung digitaler Systeme geschult sind, Entwickler ihre Wertannahmen explizit machen sowie Institutionen geeignete Prüf- und Sicherheitsmechanismen etablieren.

#### Erklärbarkeit und Rechtfertigung

Ein zentrales Problem lernender Systeme besteht darin, dass ihre Funktionsweise häufig nicht mehr vollständig erklärbar ist [5, 6]. Ärztinnen und Ärzte, die auf solche Black-Box-Modelle zurückgreifen, können ggf. nicht im Detail nachvollziehen, warum ein System eine bestimmte Empfehlung oder Beurteilung trifft – und sind folglich kaum in der Lage, diese gegenüber Patientinnen und Patienten erschöpfend zu vermitteln. Damit wird nicht nur das Recht auf informierte Zustimmung, sondern auch die Grundlage ärztlicher Kommunikation sowie des Vertrauensverhältnisses zwischen Ärztinnen und Patienten berührt.

Daraus leiten manche Autorinnen und Autoren ein Recht auf menschliche bzw. nicht-KI-basierte Entscheidungsalternativen ab. Andere fordern den Einsatz sog. interpretierbarer KI, deren Entscheidungsprozesse für Fachleute nachvollziehbar bleiben sollen. In der Praxis zeigt sich jedoch, dass vollständige Erklärbarkeit bei komplexen neuronalen Netzen oder adaptiven Robotiksystemen technisch bislang oftmals nicht erreichbar ist.

Ethisch entscheidend ist hierbei die Unterscheidung zwischen Erklärung und Rechtfertigung. Nicht in jedem Fall muss verstanden werden, *wie* ein System funktioniert, wohl aber, *warum* sein Einsatz und womöglich eine darauf basierende Entscheidung gerechtfertigt ist [7, 8]. In der Medizin beruht diese Rechtfertigung auf 2 Säulen:

- empirischer Evidenz, d. h. auf dem Nachweis, dass ein Verfahren unter realen Bedingungen zuverlässig wirksam ist (vgl. unter „Beurteilung von Wirksamkeit und Zuverlässigkeit“);
- Übereinstimmung mit den Werten sowie Präferenzen der Patientinnen und Patienten (vgl. unter „Autonomie und Entscheidungsfindung“), etwa der Lebensverlängerung, der Funktionsverbesserung oder der Förderung von Lebensqualität.

Für das Vertrauen in KI und Robotik ist daher nicht primär die technische Transparenz entscheidend, sondern die ethische Nachvollziehbarkeit ihres Einsatzes, indem ihre Anwendung begründet wird, im Entscheidungsfindungsprozess überprüfbar bleibt und im Einklang mit patientenzentrierten Zielen steht [5, 7].

#### Vertraulichkeit, Privatsphäre und Datensouveränität

Der Einsatz von KI und robotischen Systemen setzt den Zugriff auf große Datenmengen voraus, bringt damit jedoch auch Risiken einer fremdzwecklichen oder missbräuchlichen Analyse und Auswertung mit sich [10–12]. Für die individuelle technologische Unterstützung werden teils hoch sensible Informationen über Gesundheitszustand, Körper und Verhalten aufgezeichnet und ausgewertet. Sensoren von robotischen Systemen wiederum erfassen kontinuierlich Bewegungs- und Leistungsdaten. Selbst bei rechtmäßiger Nutzung können normative Spannungen zwischen Datenverfügbarkeit, Schutzinteressen und informierter Datensouveränität entstehen.

Die etablierten Modelle des Datenschutzes geraten hier an ihre Grenzen. Klassische Einwilligungskonzepte sind kaum geeignet, die Vielzahl möglicher Nutzungen antizipativ abzudecken. Entsprechend werden Formen diskutiert, wie etwa ein „broad consent“, der eine allgemeine Zustimmung zu zukünftigen Forschungsnutzungen vorsieht, ein „meta-consent“, bei dem Personen selbst festlegen können, welche Arten von Datenverwendung künftiger Zustimmung bedürfen, sowie ein „dynamic consent“, der über interaktive Plattformen eine fortlaufende, situationsbezogene Einwilligung ermöglicht. Diese Modelle versuchen, das Spannungsfeld zwischen Forschungsfreiheit und individueller Kontrolle neu auszubalancieren.

Parallel dazu gewinnen technische Schutzmaßnahmen an Bedeutung. Verfahren zur Anonymisierung und Pseudonymisierung, datenschutzfreundliche Speicherstrukturen (z. B. auch Blockchain-basierte Systeme) oder zentrale Treuhandstellen sollen Vertraulichkeit sichern. Gleichwohl bleibt das Risiko der Re-Identifizierbarkeit bislang bestehen, da medizinische Datensätze häufig einzigartig sind sowie insbesondere bei Kombination bildgebender, genetischer und verhaltensbezogener Daten eine Rekonstruktion möglich machen [11, 12].

Ethisch entscheidend ist daher die Wahrung von Vertraulichkeit und Datensouveränität. Patientinnen und Patienten müssen – praktikabel und niederschwellig – nachvollziehen können, wer Zugriff auf ihre Daten hat, zu welchem Zweck diese genutzt werden und wie sie geschützt bleiben.

#### Deskilling und Dehumanisierung

Mit der zunehmenden Integration von KI und Robotik in der Medizin verbindet sich die Sorge, dass menschliche Fachkompetenz an Bedeutung verlieren und die ärztliche Rolle durch algorithmische Systeme ersetzt werden könnte [8, 9]. Diese Befürchtung reicht von einem schleichenden „deskilling“ – also dem Verlust praktischer Fertigkeiten und klinischer Urteilskraft – bis hin zur Vorstellung einer Entmenschlichung der medizinischen Praxis, in der Ärztinnen und Ärzte zu bloßen Kontrollinstanzen algorithmisch geleiteter Prozesse werden.

Tatsächlich besteht die Möglichkeit, dass fortschreitende Automatisierung jene Kompetenzen schwächt, die die ärztliche Tätigkeit traditionell auszeichnen, nämlich das Erfahrungsurteil, die narrative Anamnese und die situative Intuition im Umgang mit Unsicherheit. Wenn Diagnostik, Therapieentscheidungen oder gar kommunikative Elemente zunehmend durch automatisierte Systeme vorbereitet oder ersetzt werden, könnte dies eine Verengung des professionellen Selbstverständnisses auf Überwachung und Protokollierung technischer Prozesse zur Folge haben.

Gleichzeitig wäre es jedoch verkürzt, diese Entwicklung als unvermeidlichen Verlust zu deuten. KI und Robotik können – richtig verstanden und eingesetzt – auch eine Renaissance ärztlicher Urteilskraft ermöglichen, indem sie Routinearbeiten reduzieren, komplexe Datenmengen strukturieren und damit Freiräume für jene Formen klinischer Aufmerksamkeit schaffen, die technikresistent bleiben. Voraussetzung ist jedoch, dass Ärztinnen und Ärzte „im System verbleiben“, d. h. die Funktionsweise, Reichweite und Grenzen der eingesetzten Technologien verstehen und in der Lage sind, aus kritischer Distanz heraus mit diesen umzugehen. Eine verantwortungsvolle Implementierung von KI und Robotik verlangt daher nicht die Reduktion ärztlicher Tätigkeit, sondern die bewusste Aneignung neuer Kompetenzen an der Schnittstelle von Technik, Ethik und klinischer Praxis.

### Chancen und Nutzen des Einsatzes

#### Leistung und Präzision auf Basis großer Datenmengen

Ein wesentlicher Nutzen von KI und Robotik liegt in ihrer Fähigkeit, durch die Berücksichtigung und Verarbeitung großer, komplexer Datensätze Muster zu erkennen, die menschlicher Wahrnehmung und Analyse bislang häufig entgehen. Auf dieser Grundlage können klinische Entscheidungen mit einer sehr hohen Genauigkeit und Verlässlichkeit unterstützt werden, etwa bei der Bildanalyse, der Risikostratifizierung oder der Steuerung chirurgischer Verfahren. Beispielhaft zeigt sich dieser Zugewinn an Präzision in der Chirurgie. Robotische Systeme erweitern die technischen Möglichkeiten des menschlichen Handelns, indem sie feinmotorische Bewegungen skalieren, physiologische Tremorbewegungen ausgleichen und die operative Sicht durch 3‑D-Visualisierung verbessern. Dadurch werden minimal-invasive Zugänge und eine schonendere Gewebebehandlung möglich, was die postoperative Erholung und funktionelle Ergebnisse verbessert [3].

Ethisch bedeutsam ist diese gesteigerte Präzision, weil sie das Risiko von Fehlentscheidungen, unbeabsichtigten Nebeneffekten und Übertherapie reduziert und damit unmittelbar dem Prinzip des Nicht-Schadens dient. Zugleich realisiert ihr Einsatz möglicherweise das Wohltunsprinzip, indem er Heilungschancen zielgenauer identifiziert und optimiert. Dementsprechend kann dort, wo solche Technologien nachweislich zu sichereren und wirksameren Behandlungen führen, der Verzicht auf ihren Einsatz sogar eine Verletzung dieser Prinzipien bedeuten [7].

#### Individualisierung (und Personalisierung) der Medizin

Damit eng zusammenhängend, bieten insbesondere KI und datenbasierte Verfahren eine neue Chance im Sinne einer individualisierten Medizin, die eine Entwicklung weg von populationsbezogenen Durchschnittswerten hin zu patientenspezifischen Risikoprofilen und Therapieempfehlungen ermöglicht. Durch die Auswertung großer Datenmengen lassen sich Subgruppen und individuelle Muster identifizieren, die herkömmlichen statistischen Modellen verborgen bleiben. So kann etwa die Wahrscheinlichkeit eines Therapieansprechens, einer Nebenwirkung oder eines Rezidivs zunehmend patientenbezogen abgeschätzt werden.

Ethisch stellt diese Entwicklung einen bedeutsamen Schritt hin zur Förderung von Autonomie und Gerechtigkeit in der medizinischen Versorgung dar. Wenn Technologien in der Lage sind, Risiken, Unsicherheiten und Konfidenzintervalle differenziert auszuweisen, ermöglicht dies besser informierte Entscheidungen und eine individuellere Aufklärung. Zugleich können bisher unterrepräsentierte Gruppen gezielt berücksichtigt und dadurch strukturelle Verzerrungen reduziert werden.

Eine Personalisierung der Medizin geht noch einen Schritt weiter als eine solche technologische Verfeinerung. In ihr drückt sich eine umfassende moralische Verpflichtung zur Individualität aus, die medizinische Entscheidungen an die konkreten Bedürfnisse, aber auch an die individuellen Werte, Erfahrungen und Lebensumstände einzelner Patienten anzupassen sucht [5, 6]. Die Berücksichtigung dieser – über das medizinisch-technische hinausgehenden – Aspekte und ihre flexible Integration in den technologiegestützten Entscheidungsprozess könnten auch zu einer personalisierten Medizin beitragen.

#### Explikation von Werten und Förderung des Arzt-Patienten-Verhältnisses

Medizinisches Handeln ist immer wertegeleitetes Handeln, auch dort, wo es sich auf Daten und Algorithmen stützt. Jede Entscheidung impliziert eine Vorstellung davon, was als „gutes“ Ergebnis oder Ziel gilt: Lebensverlängerung, Lebensqualität, Sicherheit, Gerechtigkeit oder Ressourcenschonung. Der Einsatz von KI und Robotik macht solche Wertannahmen unübersehbar, weil die Bewertung ihrer Leistung immer die explizite Festlegung von Zielgrößen und Schwellenwerten erfordert (vgl. unter „Normative Setzungen der Technik und fehlende Wertflexibilität“). Ob etwa eine höhere Sensitivität zulasten der Spezifität ethisch vorzuziehen ist oder welches Risiko an Fehlklassifikationen akzeptabel bleibt, sind keine technischen Fragen, sondern Wertentscheidungen.

Damit eröffnet die Implementierung solcher Technologien die Chance, die bislang oft impliziten normativen Maßstäbe medizinischen Handelns durch deren Operationalisierung sichtbar, überprüfbar und diskutierbar zu machen. Wo diese Werte transparent gemacht werden, kann eine reflexive und partizipative Entscheidungsfindung entstehen, in der Patienten, Ärztinnen und Institutionen über Prioritäten und Zielkonflikte offen verhandeln.

In diesem Sinn kann die Integration von KI und Robotik – bei transparenter und kritischer Anwendung – das Ärztin-Patienten-Verhältnis sogar fördern, indem sie Kommunikation über Werte in den Vordergrund rückt, gemeinsame Entscheidungen fördert und Vertrauen auf der Basis von Nachvollziehbarkeit stärkt [9]. Der Einsatz technologischer Systeme wird auf diese Weise zum Anlass ethischer Klärung.

#### Erweiterung von Zugang und Gerechtigkeit

Die oben beschriebenen Risiken algorithmischer Verzerrung und Diskriminierung machen indes deutlich, welches Potenzial in KI und Robotik für die Förderung von Gerechtigkeit liegt. Richtig konzipiert und implementiert, können sie dazu beitragen, strukturelle Benachteiligungen zu verringern und medizinische Versorgung breiter zugänglich zu machen – etwa durch automatisierte Diagnostik in ressourcenarmen Regionen, telemedizinische Betreuung oder adaptive Assistenzsysteme für Menschen mit Behinderungen.

Technologien und digitale Systeme können Zugangsgerechtigkeit fördern, indem sie dabei unterstützen, Expertise zu verteilen, Versorgungslücken zu schließen sowie Qualitätsstandards unabhängig von geographischen und sozioökonomischen Bedingungen zu sichern [12, 13]. Voraussetzung ist allerdings, dass diese Technologien selbst gerecht gestaltet sind, d. h. auf repräsentativen Daten beruhen, kulturell anpassbar bleiben und infrastrukturell verfügbar gemacht werden.

#### Effizienzsteigerung und Ressourcenschonung

Ein ebenso ethisch bedeutsamer Nutzen von KI und Robotik liegt in ihrer Fähigkeit, medizinische Prozesse zu optimieren und Ressourcen gezielter einzusetzen. Automatisierte Systeme können administrative Routinen übernehmen, Abläufe koordinieren und Arbeitslasten reduzieren. Mögliche Effizienzgewinne sind nicht bloß organisatorisch relevant, sondern besitzen moralische Bedeutung, da sie den schonenden und gerechten Umgang mit begrenzten Ressourcen fördern. Zeit, Aufmerksamkeit und personelle Kapazitäten werden freigesetzt und können dorthin gelenkt werden, wo sie am meisten wertgeschätzt werden, nämlich in die direkte Zuwendung zu und die Interaktion mit Patienten [9]. Der ethische Wert besteht damit nicht vorrangig in der maximalen Leistungssteigerung und Kosteneinsparung an sich, sondern in der klugen Allokation von Mitteln im Dienst des Patientenwohls.

#### Förderung wissenschaftlicher Erkenntnis und Innovation

KI und robotische Systeme verändern schließlich nicht nur die klinische Praxis, sondern auch die Grundlagen wissenschaftlicher Erkenntnis. Ihre Fähigkeit, große und multimodale Datensätze zu analysieren, erlaubt es, bislang verborgene Korrelationen aufzudecken, Hypothesen zu generieren und hiermit Forschung zu beschleunigen – von der Molekularbiologie über die Epidemiologie bis hin zur Versorgungsforschung. In der Chirurgie ermöglichen robotische Plattformen standardisierte Datenerfassung und -auswertung, die für Qualitätskontrolle und kontinuierliches Lernen genutzt werden können.

Mit diesem Potenzial eines epistemischen Fortschritts geht auch eine ethische Dimension einher. Wissenschaftliche Erkenntnis gilt in der Medizin selbst als moralisches Gut, da sie zur Verbesserung von Behandlung, Prävention und Lebensqualität beitragen kann [5, 7]. Systeme, die Wissen effizienter und präziser erzeugen, fördern somit indirekt die Prinzipien des Wohltuns und des Nicht-Schadens. Der Erkenntnisgewinn durch KI ist aber nur dann ethisch wertvoll, wenn er auf nachvollziehbaren Methoden beruht, die eine kritische Auseinandersetzung mit ihnen ermöglichen und deren Anwendung vorrangig auf die Förderung des Patientenwohls zielt.

## Synthese: Transformation der medizinischen Praxis

Die Einführung von KI und Robotik in die Medizin verändert nicht nur die Mittel, mit denen diagnostische, therapeutische oder rehabilitative Aufgaben erfüllt werden. Sie formen die Grundstrukturen medizinischer Praxis selbst um – jene Gefüge von Wissen, Normen und Beziehungen, die das Handeln im Gesundheitswesen tragen. Diese Transformation vollzieht sich in drei eng miteinander verwobenen Dimensionen, nämlich einer epistemischen, einer normativen und einer sozialen. Jede dieser Dimensionen führt zu Fragen, die die oben genannten Chancen und Risiken zu bündeln versuchen.

### Epistemische Transformation: Wie medizinisches Wissen entsteht und begründet wird

Mit der Integration datengetriebener Systeme verschiebt sich das epistemische Fundament medizinischer Praxis. Traditionell beruht medizinisches Wissen auf der Verbindung kausal-mechanistischer Erklärungen mit empirisch geprüfter Evidenz – ein Zusammenspiel, das durch die Prinzipien der evidenzbasierten Medizin strukturiert ist [5, 6]. KI und Robotik verändern diese Ordnung, indem sie Wissen zunehmend induktiv und probabilistisch generieren. Nicht mehr die theoretisch begründete Hypothese steht am Anfang, sondern die algorithmische Mustererkennung in großen Datensätzen. Dadurch treten neue Formen epistemischer Autorität hervor – solche, die weniger auf nachvollziehbaren Kausalmechanismen als auf statistischer Performanz beruhen. Wissen wird so zu einem Ergebnis technischer Lernprozesse, dessen Geltung sich eher aus Prognosekraft als aus theoretischer Begründung speist. Diese Verschiebung fordert das ärztliche Selbstverständnis heraus, weil sich Begründung, Vertrauen und Rechenschaft zunehmend zwischen menschlicher Urteilskraft und algorithmischer Performanz verteilen [5–7].

In der Praxis der HNO manifestiert sich dies etwa in der Nutzung von Radiomics. Bildgebende Verfahren (z. B. CT, MRT) werden durch Algorithmen ergänzt, die Texturen, Grauwerte und Formmerkmale erfassen, die für das menschliche Auge kaum wahrnehmbar sind, und daraus Prognosen über Tumorverhalten oder Therapieerfolg ableiten. Aus diesem Grund ist zugleich aber der Weg, wie die Prognose konkret zustande kommt, oft nicht vollständig nachvollziehbar [5, 6].

Ethisch wirft dies mehrere Fragen auf:

- In welchem Maße ist Nachvollziehbarkeit (Transparenz) erforderlich, damit Ärztinnen und Ärzte und Patientinnen und Patienten wissen, worauf eine Entscheidung beruht und aus welchen Gründen eine darauf aufbauende Entscheidung gerechtfertigt ist?
- Welche epistemische Verpflichtung besteht, dass Wissen begründbar bleibt und dass man Fehlerquellen identifizieren kann?
- Wie wirkt sich diese Verschiebung auf das Selbstverständnis ärztlicher Urteilskraft aus – insbesondere, wenn KI-Systeme Wahrscheinlichkeiten liefern, wo zuvor Erfahrung und heuristische Methoden dominierten?

### Normative Transformation: Veränderung dessen, was als gute medizinische Entscheidung gilt

Die normative Dimension der Transformation betrifft die Frage, was in der Medizin als moralisch gute, verantwortliche oder gerechte Entscheidung gilt. Seit der Etablierung der modernen Medizinethik in den 1970er-Jahren wurde ärztliches Handeln wesentlich durch Prinzipien wie Wohltun, Nicht-Schaden sowie Respekt vor Autonomie und Gerechtigkeit geleitet, ergänzt durch den Anspruch einer evidenzbasierten und patientenzentrierten Praxis [7, 10]. KI und Robotik führen hier keine gänzlich neuen Werte ein, wohl aber verändern sie deren Gewichtung und Verhältnis. Effizienz, Präzision und Risikominimierung gewinnen normative Bedeutung, während Erfahrungswissen, klinische Urteilskraft oder narrative Dimensionen medizinischer Begegnung an Sichtbarkeit verlieren können [6]. Damit verschiebt sich der Ort ethischer Bewertung – vom individuellen Urteil hin zu modellierten, technisch vermittelten Entscheidungsräumen.

Beispielhaft zeigt sich dies bei der Behandlung von Kopf-Hals-Tumoren. Algorithmische Systeme können Behandlungsvorschläge erzeugen, die entweder die Lebenserwartung oder die funktionelle Erhaltung von Stimme oder Schluckfähigkeit priorisieren. Werden solche Priorisierungen implizit durch technische Systeme vorgenommen, ohne dass sie mit individuellen Wertvorstellungen oder patientenseitigen Präferenzen abgestimmt sind, entstehen normative Spannungen [5, 7, 9].

Ethisch wirft dies zentrale Fragen auf:

- Welche Werte werden in den Systemen kodiert, und wie lassen sich Zielkonflikte transparent und verhandelbar machen?
- Wie können Patienten in technisch gestützte Entscheidungen einbezogen werden, um ihre Präferenzen hinreichend zu berücksichtigen?
- Oder anders: Wie gelingt die gemeinsame Entscheidungsfindung, wenn der Technologieeinsatz wahrgenommene oder reale Handlungsräume modifiziert?

Normative Transformation bedeutet damit nicht vorrangig die Einführung neuer Werte, sondern v. a. die Neubewertung bereits bestehender Werte. Sie macht deliberative Verfahren erforderlich, die reflektierte Abwägungen und Transparenz ermöglichen.

### Soziale Transformation: Rollen, Beziehungen und institutionelle Akteure im Wandel

Die soziale Dimension betrifft die Struktur und Dynamik medizinischer Praxis als kollektiven und institutionellen Prozess. KI und Robotik sind keine neutralen Werkzeuge, sondern Akteure in soziotechnischen Systemen, die Interaktionen, Zuständigkeiten und Autorität neu ordnen [6, 9, 11]. Ärztinnen und Ärzte, Patientinnen und Patienten, Institutionen, Entwickler und Regulatoren agieren zunehmend in hybriden Netzwerken, in denen menschliche und technische Beiträge verschränkt sind. Damit verändern sich nicht nur Handlungsabläufe, sondern auch die soziale Ordnung des medizinischen Handelns: Verantwortung, Vertrauen und Entscheidung werden geteilt [8–11] – mit Systemen, deren Beiträge zwar nicht intentional, aber doch wirkmächtig sind.

In der Praxis der HNO-Chirurgie zeigt sich dies z. B. beim Einsatz robotischer Assistenzsysteme. Operateurinnen und Operateure arbeiten gemeinsam mit robotischen Instrumenten, wobei Verantwortung – nicht jedoch Haftung – geteilt, aber nicht immer als eindeutig zugeordnet wahrgenommen wird [8]. Gleichzeitig verändert sich womöglich die Stellung oder Rolle von Patientinnen und Patienten im Rahmen der Entscheidungsfindung. Algorithmisch generierte Risikoabschätzungen und Prognosen erhöhen Erwartungen an Aufklärung, Mitsprache und informierte Zustimmung [9, 11]. Institutionell sind Kliniken, Hersteller und Zulassungsbehörden gefordert, die Nutzung der Systeme zu überwachen, Haftungsfragen zu klären und Standardisierungsprozesse zu implementieren [8, 12].

Ethisch wirft dies etwa die folgenden Fragen auf:

- Wie lässt sich Verantwortung in hybriden Mensch-Maschine-Konstellationen transparent gestalten?
- Wie können Vertrauen und partizipative Entscheidungsprozesse trotz zunehmender Technologisierung aufrechterhalten werden?
- Wie lässt sich sicherstellen, dass institutionelle und gesellschaftliche Rahmenbedingungen den Schutz der Patientinnen und Patienten sowie die Qualität der Versorgung gewährleisten?

Soziale Transformation bedeutet damit die Neuaushandlung professioneller Rollen, institutioneller Verantwortlichkeiten und gesellschaftlicher Erwartungen im Gesundheitswesen.

## Ethische Praxis: Von Prinzipien zum verantwortungsvollen Einsatz

Diese dreifache Transformation – epistemisch, normativ und sozial – markiert nicht nur eine theoretische Verschiebung, sondern eine Phase, in der die Bedingungen medizinischer Praxis neu verhandelt werden. Damit verändern sich mit dem Einsatz der Technologien nicht nur die genutzten Werkzeuge, sondern auch die Erwartungen, Verpflichtungen und Rollen in der Medizin.

Die ethische Diskussion über KI und Robotik in der Medizin hat in den vergangenen Jahren eine beeindruckende Breite erreicht. Zahlreiche Positionspapiere, Leitlinien und Frameworks – etwa jene der Europäischen Kommission [14] oder der World Health Organization (WHO) [15], aber auch solche der Zentralen Ethikkommission bei der Bundesärztekammer [12] oder des Deutschen Ethikrats [13] – formulieren oftmals Grundprinzipien und Rahmenbedingungen eines verantwortungsvollen Einsatzes v. a. von KI (vgl. zum internationalen Überblick auch v. a. [16]). Diese Prinzipien bilden einen wichtigen normativen Ausgangspunkt. Doch die Formulierung von oder die Berufung auf allgemeine Prinzipien ist nicht gleichbedeutend mit ihrer praktischen Wirksamkeit. In der klinischen und institutionellen Realität zeigt sich, dass Prinzipien *eo ipso* häufig zu abstrakt bleiben, um Anschaffungsentscheidungen, Implementierungsprozesse oder konkrete Behandlungsentscheidungen von Anwenderinnen und Anwendern tatsächlich zu leiten. Der entscheidende Schritt besteht daher darin, die Ethik von KI und Robotik prozessual zu verstehen [18, 22, 23] – als kontinuierliche Praxis, die sich in situativem Handeln, organisatorischen Routinen und interdisziplinären Aushandlungen realisiert und in diesen die oben genannten Prinzipien zu realisieren und auszuhandeln sucht.

Eine solche prozess- und kontextgeleitete Ethik verschiebt den Fokus von der Frage, welche Prinzipien gelten, zu der Frage, wie ethische Maßstäbe in konkreten Abläufen verwirklicht werden. Sie verlangt Verfahren, die ethische Reflexion in Beschaffung, Implementierung und Anwendung einbetten, etwa durch interdisziplinäre Evaluationsgremien, adaptive Risikoabwägung, partizipative Entscheidungsprozesse und kontinuierliche Rückkopplungsschleifen zwischen klinischer Erfahrung und technischer Entwicklung.

Ein verantwortungsvoller Einsatz von KI und Robotik muss daher reflexiv, dialogisch und iterativ lernfähig sein. Dies erfordert ein Verständnis der kontinuierlichen ethischen Deliberation als Bestandteil technischer und klinischer Praxis, wie dies unterschiedliche Ansätze einer entwicklungs- und anwendungsnahen Ethik versuchen (z. B. Embedded Ethics; [18, 24]). Das schließt etwa ein, dass Leitlinien regelmäßig überarbeitet werden, dass empirische Untersuchungen des Technologieeinsatzes neben medizinisch-technischen auch patientenzentrierte Outcomes erheben oder dass unvorhergesehene Folgen (z. B. neue Bias-Effekte, Fehlinterpretationen oder negative Auswirkungen auf das Verhältnis von Gesundheitsfachpersonen und Patientinnen und Patienten) systematisch dokumentiert und öffentlich zugänglich gemacht werden. Nur so kann der Einsatz dieser Technologien nicht nur technisch sicher, sondern auch ethisch tragfähig werden.

## Ethische Leitideen für den verantwortungsvollen Einsatz

Der Schritt von Prinzipien zu Prozessen erleichtert eine angemessene Perspektive. Ethik wird dabei nicht länger als externe Beurteilungsinstanz missverstanden, sondern als integraler Bestandteil medizinischer Praxis und Innovation. Verantwortlicher KI- und Robotikeinsatz bedeutet demnach nicht nur, ethische Anforderungen zu erfüllen, sondern ethische Reflexion in den Prozess technischer und klinischer Praxis einzuschreiben und dank dieses Prozesses bereits vielen ethischen Anforderungen zu genügen. Im Folgenden werden drei Leitideen skizziert, die eine solche Ethik des verantwortungsvollen Einsatzes von KI und Robotik in der Medizin praktisch tragen können und die mir besonders relevant erscheinen:

- epistemische Transparenz,
- geteilte Verantwortung,
- diskursiv-prozedurale Legitimation.

Diese drei Leitideen sind keine isolierten Normen, sondern reflexive und iterative Prinzipien, die sich in Interaktion entfalten sowie damit ein Grundgerüst einer lernenden, dialogischen und ethisch fundierten Praxis darstellen können.

### Epistemische Transparenz

Epistemische Transparenz bezeichnet nicht bloß technische Nachvollziehbarkeit, sondern vielmehr eine ethische Haltung des Anspruchs an die Erzeugung wissenschaftlich und demokratisch zugänglichen Wissens, also das Bemühen, Entscheidungsprozesse so offenzulegen, dass sie begründbar, überprüfbar und kritisierbar bleiben [5, 6]. In der medizinischen Praxis bedeutet dies, dass KI-Systeme nicht vollständig „erklärbar“ im mathematisch-technischen Sinne sein müssen, wohl aber in ihren Annahmen, Grenzen und Unsicherheiten für die externe Beurteilung des technischen Systems und seiner Empfehlungen transparent gemacht werden (vgl. [25]). Dazu gehören z. B.:

- dokumentierte Trainings- und Validierungsdaten;
- evaluierte und zugänglich gemachte Fehlerquoten;
- klare Darlegung der Zweckbestimmung und des bestimmungsgemäßen Gebrauchs;
- Mechanismen und Umgangsstrategien, um mögliche Diskrepanzen zwischen einer Technologie und dem klinischen Urteil ihrer Anwenderinnen und Anwender systematisch zu erfassen.

Ethisch geht es dabei um etwas, das mitunter als epistemische Integrität (vgl. [26, 27]) bezeichnet wurde. Ärztinnen und Ärzte dürfen Entscheidungen nicht delegieren, ohne das epistemisch berechtigte Vertrauen in die zugrunde liegende Evidenz prüfen zu können [5, 6]. Transparenz ist deshalb nicht nur eine Frage der Architektur von Informationen und Wissen, sondern eine nach der Ermöglichung ärztlicher Urteilskraft und praktischer Rechenschaft trotz der Unterstützung durch digitale Technologien [7, 8].

Im Falle der HNO zeigt sich diese Herausforderung exemplarisch. KI-basierte Tumorgradierung oder Sprachanalyse-Tools können klinisch sehr nützlich sein, aber ihre Vertrauenswürdigkeit hängt davon ab, ob nachvollziehbar – und damit auch bei Bedarf modifizierbar – bleibt, auf welchen Merkmalen ihre Empfehlungen beruhen und mit welchen Limitationen diese anzuwenden sind.

### Geteilte Verantwortung

Die zweite Leitidee betrifft „neue“ Formen kollektiven Handelns, die durch KI und Robotik gehäuft auftreten – und damit an Diskursen zum „problem of many hands“ [28] oder zu Verantwortungslücken [29] anknüpfen. Auch medizinische Entscheidungen werden zunehmend in hybriden Systemen und durch Interaktionen zwischen Menschen und Technologien getroffen. Ärztinnen, Entwickler, Institutionen, regulatorische Instanzen und Hersteller sind zunehmend gemeinsam beteiligt.

Ethisch kann Verantwortung hier nicht einfach individuell zugeschrieben werden [8]; sie muss strukturiert geteilt werden, auch wenn diese Tatsache womöglich in einem Spannungsverhältnis zur ärztlichen Pflicht der (Letzt‑)Aufsicht über den gesamten Behandlungsprozess stehen mag [12]. Exemplarisch zeigt sich dieser Bedarf im Falle der robotisch-assistierten HNO-Chirurgie. Wenn intraoperative Entscheidungen auf Basis algorithmischer Vorschläge getroffen werden, muss geklärt sein, wer in welchem Moment die Kontrolle hat und damit in der Regel die moralische Verantwortung und die rechtliche Haftung übernimmt – der Operateur, das System oder die Institution, die das System bereitstellt.

Das Ziel sollte demnach eine Verantwortungsarchitektur sein, die Verantwortlichkeit nicht verteilt, um sie auszudünnen oder zu verschleiern, sondern um die Frage nach Verantwortlichkeit adressierbar zu machen (vgl. auch [30]). Geteilte Verantwortung bedeutet hierbei begründete und nachvollziehbarer Rechenschaft. Eine solche Architektur könnte etwa die folgenden Rollen vorsehen:

- Entwicklerinnen und Entwickler, welche Verantwortung für Datenqualität, Modellarchitektur und Bias-Prüfung tragen;
- Gesetzgeber, Behörden und Institutionen für Regulierung/Governance, Prüfung und Risikomanagement;
- Fachgesellschaften und Kliniken für fachliche Evaluation und Auswahl der Systeme sowie für Angebote zur Schulung;
- Ärztinnen und Ärzte für den reflektierten und kompetenten Einsatz im individuellen Fall.

Dem liegt ethisch die Idee der individuellen Zumutbarkeit zugrunde. Verantwortung darf nur dort verlangt werden, wo reale Handlungsmöglichkeiten bestehen („*ultra posse nemo tenetur*“). D. h., eine Ärztin kann nicht für die innere Logik eines geschlossenen Systems verantwortlich sein, wohl aber für die Entscheidung, es fachlich angemessen einzusetzen (vorausgesetzt, sie ist dazu in die Lage versetzt; vgl. „Epistemische Transparenz“; [8]).

### Diskursiv-prozedurale Legitimation

Die dritte Leitidee betrifft die Frage, wie normative Entscheidungen über den Einsatz von KI und Robotik zustande kommen und gerechtfertigt werden. Ethische Legitimität erwächst – zumindest diskursethischen Ansätzen folgend – nicht aus technischer Performanz oder formaler Regelbefolgung, sondern aus kommunikativer Aushandlung, also aus der Möglichkeit, Entscheidungen gegenüber Dritten oder öffentlich zu begründen, zu prüfen und ggf. zu revidieren.

Für die medizinische Praxis könnte dies bedeuten, dass Fragen nach Einführung, Nutzung und Weiterentwicklung neuer Technologien nicht exklusiv in Expertengremien oder Verwaltungsstrukturen verbleiben dürfen. Stattdessen bedürfen Entscheidungen über Systeme, die die Wahrnehmung von Krankheit, Autonomie und sozialer Teilhabe prägen, einer breiten und pluralen Deliberation. Patientinnen und Patienten, Behandlerinnen und Behandler, Entwicklerinnen und Entwickler wie auch gesellschaftliche Akteure sollten in Verfahren eingebunden werden, die divergierende Perspektiven sichtbar machen und ausbalancieren.

Diskursiv-prozedurale Legitimation zielt dabei auf eine begründete Zustimmung im Dialog. Sie verlangt, dass die zugrunde liegenden Wertentscheidungen – etwa zu Prioritäten zwischen Sicherheit, Effizienz, Zugänglichkeit oder Gerechtigkeit – öffentlich und argumentativ nachvollziehbar gemacht werden. Gerade dort, wo KI- oder Robotiksysteme Wertabwägungen implizit in algorithmischen Gewichtungen kodieren, muss die Möglichkeit bestehen, diese Gewichtungen zur Diskussion zu stellen und ggf. zu korrigieren. So verstanden, lassen sich diskursiv-prozedurale Legitimation und epistemische Transparenz (vgl. oben) als komplementäre Voraussetzungen von Vertrauen in technologische Systeme begreifen. Sie ermöglichen, dass Vertrauen in KI und Robotik nicht blind oder erzwungen, sondern durch nachvollziehbare Gründe verdient wird – durch Verfahren, die Rechenschaft, Partizipation und kritische Überprüfbarkeit institutionalisieren.

Eine solche diskursiv-prozedurale Legitimation ist freilich nicht ohne institutionelle und gesundheitspolitische Implikationen. Sie setzt voraus, dass ethische Deliberation nicht als freiwillige Ergänzung, sondern als Bestandteil von Governance und Qualitätsmanagement im Gesundheitssystem etabliert wird. Dies erfordert Strukturen der Partizipation, eine Bereitschaft der kontinuierlichen Evaluation und Revision von gescheiterten Implementierungsversuchen sowie die Anerkennung von Dissens und Perspektivenvielfalt als Ausdruck professioneller Rechenschaft und nicht als Hindernis effizienter Innovation.

## Schluss und Ausblick

Die Diskussion um KI und Robotik in der Medizin befindet sich – nach wie vor – an einem entscheidenden Punkt. Die technologischen Entwicklungen schreiten mit einer Geschwindigkeit voran, die klinische Innovation und ethische Reflexion in ein Spannungsverhältnis zu bringen scheint. Die entscheidende Frage ist längst nicht mehr, ob solche Technologien grundsätzlich eingesetzt werden sollen, sondern wie ihr Einsatz gestaltet werden kann, damit sie den Prinzipien einer patientenzentrierten und verantwortlichen Medizin entsprechen.

Einerseits eröffnen KI-gestützte Systeme und robotische Verfahren Möglichkeiten, die die Qualität, Präzision und Zugänglichkeit medizinischer Versorgung erheblich erweitern können. Andererseits transformiert ihr Einsatz die Grundlagen medizinischer Praxis – die Entstehung von Wissen, die Kriterien des moralisch guten Handelns und die sozialen Beziehungen, auf denen medizinische Vertrauens- und Verantwortungsstrukturen beruhen.

Die ethische Aufgabe aller am Prozess Beteiligten besteht nicht mehr allein darin, diese Veränderungen zu bewerten, sondern sie mitzugestalten. Verantwortungsvoller Einsatz heißt dabei, die moralischen Voraussetzungen der Medizin – Prinzipien wie Wohltun und Nicht-Schaden, Respekt vor der Autonomie, Gerechtigkeit, aber auch Werte wie Würde und Wahrhaftigkeit – nicht gegen technologische Innovationen auszuspielen, sondern sie diesen einzuschreiben. Ethische Prinzipien bleiben damit zwar grundlegend, doch erst in prozessgeleiteten Strukturen – durch epistemische Transparenz, geteilte Verantwortung und diskursive Legitimation – gewinnen sie reale Wirksamkeit.

Die Ethik steht hierbei nicht als Instanz außerhalb, sondern als Akteurin mitten im Prozess. Ihre Aufgabe ist es, das Gespräch offen zu halten und zur Verständigung beizutragen: zwischen technischen Möglichkeiten und menschlichen Bedürfnissen, zwischen systemischer Innovation und individueller Verantwortung. Verantwortungsvoll mit KI und Robotik umzugehen bedeutet deshalb nicht nur, Risiken zu minimieren, sondern die Bedingungen zu sichern und zu schaffen, unter denen Medizin eine soziale und moralische Praxis bleibt.

## Fazit für die Praxis

- Künstliche Intelligenz (KI) und Robotik sind nicht nur neue Werkzeuge, sondern sie verändern die Strukturen der medizinischen Praxis selbst – Wissen, Entscheidungsmaßstäbe und Verantwortungsbeziehungen.
- Verantwortlicher Einsatz bedeutet nicht technologische Zurückhaltung, sondern die aktive Gestaltung von Bedingungen, unter denen Medizin menschlich, gerecht und vertrauenswürdig bleibt.
- Ethische Prinzipien wie Wohltun, Nicht-Schaden, Gerechtigkeit und Respekt vor der Autonomie bleiben zentral, müssen aber in konkrete Prozesse und Institutionen übersetzt werden.
- Epistemische Transparenz ist Voraussetzung für begründetes Vertrauen. Systeme müssen in ihren Annahmen, Zielsetzungen, Grenzen und Unsicherheiten nachvollziehbar sein.
- Verantwortung im Technologieeinsatz ist geteilt und erfordert klare Zuständigkeiten und Rechenschaftswege.
- Legitimation des Einsatzes neuer Technologien muss diskursiv erfolgen – durch partizipative, pluralistische Entscheidungsprozesse, die divergierende Werte sichtbar machen.
